# Stable high frequencies of sulfadoxine–pyrimethamine resistance associated mutations and absence of K13 mutations in *Plasmodium falciparum* 3 and 4 years after the introduction of artesunate plus sulfadoxine–pyrimethamine in Ujjain, Madhya Pradesh, India

**DOI:** 10.1186/s12936-020-03274-w

**Published:** 2020-08-14

**Authors:** Ashish Pathak, Andreas Mårtensson, Sudhir Gawariker, Ashish Sharma, Vishal Diwan, Manju Purohit, Johan Ursing

**Affiliations:** 1 Department of Pediatrics, R D Gardi Medical College, Surasa, 456010 Ujjain, India; 2 Department of Women and Children’s Health, International Maternal and Child Health Unit, Uppsala University, 751 85 Uppsala, Sweden; 3 Global Health–Health Systems and Policy: Medicines, Focusing Antibiotics, Department of Global Public Health, Karolinska Institutet, Tomtebodavägen 18A, 171 77 Stockholm, Sweden; 4 Department of Medicine, R D Gardi Medical College, Surasa, 456010 Ujjain, India; 5 Public Health & Environment in R D Gardi Medical College, Ujjain, India; 6 Department of Pathology, R D Gardi Medical College, Surasa, 456010 Ujjain, India; 7 Department of Infectious Diseases, Danderyd Hospital, Stockholm, Sweden; 8 Department of Clinical Sciences, Karolinska Institutet, Danderyd Hospital, Stockholm, Sweden

**Keywords:** *Plasmodium falciparum*, Artesunate sulfadoxine pyrimethamine, Chloroquine, Resistance, *pfcrt*, *pfmdr1*, *pfdhfr*, *pfdhps*, *pfnhe1*, *K13*

## Abstract

**Background:**

Artesunate plus sulfadoxine–pyrimethamine (ASP) is first-line treatment for uncomplicated *Plasmodium falciparum* malaria in most of India, except for six North-eastern provinces where treatment failure rates were high. In Ujjain, central India, the frequency of mutations associated with increased drug tolerance, but not overt resistance to sulfadoxine and pyrimethamine were 9% and > 80%, respectively, in 2009 and 2010, just prior to the introduction of ASP. The frequency of drug resistance associated mutations in Ujjain in 2015–2016 after 3–4 years of ASP use, are reported.

**Methods:**

Blood samples from patients with *P. falciparum* mono-infection verified by microscopy were collected on filter-paper at all nine major pathology laboratories in Ujjain city. Codons *pfdhfr* 16–185, *pfdhps* 436–632 and K13 407–689 were identified by sequencing. *Pfcrt* K76T and *pfmdr1* N86Y were identified by restriction fragment length polymorphism.

**Results:**

Sulfadoxine–pyrimethamine resistance-associated *pfdhfr* 108 N and 59R alleles were found in 100/104 (96%) and 87/91 (96%) samples, respectively. *Pfdhps* 437G was found in 10/105 (10%) samples. Double mutant *pfdhfr* 59R + 108 N were found in 75/81 (93%) samples. Triple mutant *pfdhfr* 59R + 108 N and *pfdhps* 437G were found in 6/78 (8%) samples. Chloroquine-resistance-associated *pfcrt* 76T was found in 102/102 (100%). *Pfmdr1* N86 and 86Y were identified in 83/115 (72%) and 32/115 (28%) samples, respectively.

**Conclusion:**

The frequency of *P. falciparum* with reduced susceptibility to sulfadoxine–pyrimethamine remained high, but did not appear to have increased significantly since the introduction of ASP. No polymorphisms in K13 associated with decreased artemisinin susceptibility were found. ASP probably remained effective, supporting continued ASP use.

## Background

Malaria caused an estimated 9.6 million infections and 16,700 deaths in India in 2017. Approximately 12% (163 million) and 81% (1.1 billion) of Indians live in high and low malaria transmission areas, respectively. Only 7% (88 million) live in areas considered to be malaria-free. Malaria thus remains a major cause of morbidity and mortality and threatens the majority of the population [1]. Moreover, malaria treatment is hampered by the spread and development of drug resistance in India as elsewhere in the world [2–4].

Chloroquine resistance arose in Southeast Asia and spread through India and Pakistan via the Northeastern states. Due to widespread chloroquine resistance, sulfalene-pyrimethamine followed by sulfadoxine–pyrimethamine (SP) monotherapy were officially recommended in Northeastern India from 1982. SP was subsequently replaced by artesunate plus sulfadoxine–pyrimethamine (ASP) in 2005, but this recommendation was discontinued in 2013 due to high treatment failure frequencies [3]. Artemether-lumefantrine (AL) is now recommended in several Northeastern states as well as in neighbouring Bangladesh. Due to spreading chloroquine resistance most of the remainder of India adopted ASP as first-line treatment by 2012. ASP is similarly recommended in neighbouring Pakistan [5]. There is thus considerable regional artemisinin and SP drug pressure and consequently a high risk of resistance spreading further. Continuous monitoring of ASP efficacy is therefore necessary [3].

Determining the frequency of genetic markers of SP resistance and reduced susceptibility to artemisnins is a rapid and easy way to monitor resistance. SP resistance is associated with mutations in the *Plasmodium falciparum* dihydrofolate reductase (*pfdhfr*) and dihyropteroate synthase (*pfdhps*) genes [6–11]. Accumulation of mutations in *pfdhfr* and *pfdhps* gradually increase the tolerability of *P. falciparum* to pyrimethamine and sulfadoxine, respectively [12]. Similarly, mutations in the Kelch 13 (K13) propeller domain of *P. falciparum* are associated with delayed parasite clearance during treatment with artemisnin derivatives. Specific genotypes in the chloroquine resistance transporter gene (*pfcrt*) and multidrug resistance gene (*pfmdr 1*) are associated with reduced susceptibility to lumefantrine [13–17].

The frequency of genotypes associated with SP and lumefantrine susceptibility in Ujjain, Madhya Pradesh, central India in 2009 and 2010, just prior to the introduction of ASP were reported previously [18]. The aim of this study was to determine the frequency of genotypes associated with reduced susceptibility to ASP and lumefantrine from the same area in samples collected 2015–2016, i.e. after 3–4 years of ASP use.

## Methods

### Study site and period

Ujjain district is located in the western part of Madhya Pradesh, central India. The population of the district is 1.9 million as per 2011 Census [19]. The climate is tropical and transmission may occur throughout the year provided the relative humidity levels support the vector survival. Ujjain district has low transmission of malaria with an annual parasite index (API) < 0.1 [20, 21]. Peak malaria transmission occurs during the warm and humid months of July to September. Data collection was from June to October in 2009, 2010, 2015 and 2016. Data from 2009 and 2010 were published previously and these samples used for K13 analyses only in the current study.

### Recruitment of patients and sample collection

Samples and data were collected by the nine major pathology laboratories located in Ujjain city, Madhya Pradesh, India. Individuals or groups of pathologists reported the results from participating laboratories. Laboratories used microscopy to examine peripheral blood smears or rapid diagnostic tests (RDT) for the diagnosis of *P. falciparum* malaria. Inclusion criteria were microscopically or RDT verified malaria. For all patients that were smear positive for malaria, a drop of blood was put onto filter papers (Whatmann™ 3MM). RDTs were collected from patients in whom RDT only was used to diagnose malaria. RDTs or filter papers were labelled with the patient’s age and gender, dried and then placed inside individual sealed plastic bags.

### Sample storage, DNA extraction

Filter papers and RDTs were stored at room temperature. DNA was extracted from two 3 mm Ø punches obtained from the filter paper or the whole RDT strips. DNA was extracted with Chelex^®^100 resin (Bio-Rad Laboratory, Hercules, CA) using the boiling method with minor modifications from the original protocol using 0.2% saponin/phosphate-buffered saline and 10% Chelex [22]. DNA was stored at − 20 °C until use.

### Molecular analyses

Amino acid positions *pfdhfr* 16–185, *pfdhps* 436–632 and *k13* 407–689 were amplified using previously described PCR protocols and then sequenced commercially [13, 15, 23–26]. The Sequencher™ software version 4.6 (Gene Codes Corporation, Ann Arbor, MI, USA) was used for sequence analysis. The *P. falciparum* 3D7 clone sequences obtained from NCBI database were used as references for *pfdhfr*, *pfdhps* and *k13*.

Previously described multiplex PCR–RFLP (restriction fragment length polymorphism) methods with minor modifications were used to identify *pfdhfr* N51I, C59R and S108N and *pfdhps* S436F/A, A437G and K540E when sequencing failed and *pfcrt* K76T and *pfmdr1* N86Y SNPs [23, 27, 28].

PCR and restriction products were resolved on 2% agarose gels (Amresco, Solon, OH, USA). All gels were stained with a nucleic acid gel stain (GelRed™, Biotium Inc, Hayward, CA, USA) and visualized under UV transillumination (GelDoc^®^, Biorad, Hercules, CA, USA). PCR products were purified and sequenced commercially (Macrogen Inc, Seoul, Korea).

### Statistical analyses

The exact incidence of *P. falciparum* malaria was not known and we therefore decided to collect as many samples as possible over a two-year period. SNP frequencies were calculated by dividing the number of SNPs by the number of patients in whom a certain the allele could be identified. Allele frequencies in 2015 and 2016 were compared using Chi squared tests.

### Ethics

Patients with uncomplicated malaria were enrolled and samples collected after informed oral consent of the patient or in the case of minors informed proxy-consent of their parent or guardian. The study was approved by the Institutional Ethics Committee of R D Gardi Medical College in Ujjain, Madhya Pradesh, India (61/2009 and 494/2016) and the Regional Ethics Committee in Stockholm, Sweden (2011/832-32/2).

## Results

A total of 127 samples were collected during the 2015 (n = 48) and 2016 (n = 79) peak malaria transmission seasons. *Plasmodium falciparum* mono infection was identified in 124 samples and *P. falciparum* + *Plasmodium vivax* double infection were found in three samples. The samples were collected from 59 females and 68 males. The median age was 25 years, range 6 months to 76 years with no significant difference between females and males. Patients came from the following district Agar (n = 6), Badnagar (n = 2), Bhopal (n = 1), Dewas (n = 3, Indore (n = 2), Jhanua (n = 2), Mahidpur (n = 4), Rajgad (n = 1), Ratlam (n = 28), Shajapur (n = 8), Ahyamgad (n = 1), Tarana (n = 6) and Ujjain (n = 59). The district was not recorded for 3 patients. Blood for DNA extraction was available from filter-papers (n = 92) or rapid diagnostic tests (n = 27).

### PCR success rate

The PCR success rate for each gene is shown in Table 1. PCR success was significantly (p < 0.001) lower if DNA was extracted from RDTs compared to filter-papers for all genes irrespective of whether RFLP or sequencing was used to identify SNPs. The difference was greatest for *pfdhfr* and *pfdhps* for which sequencing was only successful in 17–20% of RDT samples compared to 88 and 89% of filter-paper samples. Overall PCR success rate ranged from 69 to 91%.

**Table 1:** Frequency of genotyping success in samples collected on filter-paper versus rapid diagnostic tests

	Number (percent) of samples with successful genotyping			Fisher’s exact p value comparing filter-paper and RDT extracted samples
	All samples (n = 127)	Filter-paper samples (n = 92)	Rapid diagnostic tests (n = 35)	
*Pfcrt* K76T	102 (80%)	87 (95%)	15 (43%)	< 0.001
*Pfmdr1* N86Y	115 (91%)	89 (97%)	26 (74%)	< 0.001
*Pfdhfr* seq	87 (69%)	81 (88%)	6 (17%)	< 0.001
*Pfdhps* seq	89 (70%)	82 (89%)	7 (20%)	< 0.001
*Pfdhfr* seq or RFLP 108	104 (82%)	84 (91%)	20 (57%)	< 0.001
*Pfdhps* seq or RFLP 436, 437, 540	105 (83%)	84 (91%)	21 (60%)	< 0.001
K13	87 (69%)	73 (79%)	14 (40%)	< 0.001

### *Pfdhfr* and *Pfdhps* SNP and haplotype frequencies

The number and frequencies of resistance-associated SNPs and haplotypes are shown in Tables 2 and 3, respectively. Double mutant *pfdhfr* 59R + 108 N were found in 78/87 (90%) samples. Triple mutant *pfdhfr* 50R + 59R + 108 N were found in 4/87 (5%) samples and double or triple were found in 82/87 (95%) samples.

**Table 2:** The number and frequency of resistance-associated *pfdhfr* and *pfdhps* alleles in *Plasmodium falciparum* samples collected in Ujjain, Madhya Pradesh, India 2015–2016

*Pfdhfr*	A16V	C50R	N51I	C59R	S108N	I164L
*Number*	87	87	91	91	104	87
Sensitive	87	83 (95%)	91 (100%)	4 (4%)	4 (4%)	87 (100%)
Resistant	0	4 (5%)	0	87 (96%)	100 (96%)	0

*Pfdhps*	S436F		A437G	K540E	A581G	A613T
Number	106		105	110	93	92
Sensitive	0		95 (90%)	110	93	92
Resistant	106		10 (10%)	0	0	0

**Table 3:**
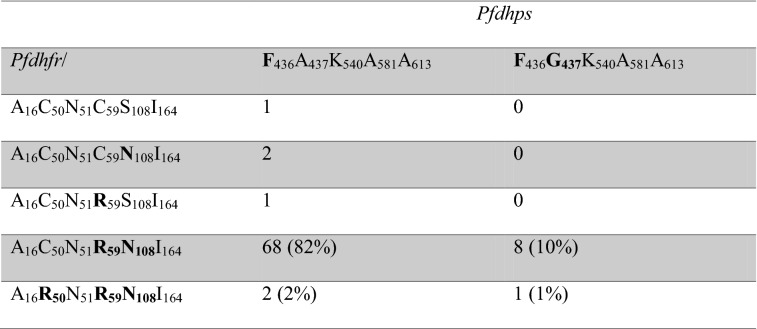
The number and frequency of *pfdhfr* and *pfdhps* resistance-associated haplotypes in *Plasmodium falciparum* samples collected in Ujjain, Madhya Pradesh, India in 2015 and 2016

The complete haplotype was available from 83 samples. The complete *pfdhps* haplotype only was FAKAA in a further 7 samples and F**G**KAA in one sample. The *pfdhfr* haplotype only was ACN**RN** and A**R**N**RN** in two and one samples, respectively

Both *pfdhfr* and *pfdhps* haplotypes were successfully sequenced in 81 samples and triple mutant *pfdhfr* 59R + 108 N and *pfdhps* 437G were found in 8/83 (10%) samples. Quadruple mutant *pfdhfr* 50R + 59R + 108 N plus *pfdhps* 437G was found in 1/83 (1%) of samples. All these haplotypes also had the 436F allele, probably adding one resistance-associated SNP to each haplotype. No sample had resistance-associated alleles at *pfdhfr* codons 51 or 164 nor at *pfdhps* codons 540, 581 or 613.

The four samples with triple mutant *pfdhfr* 50R + 59R + 108 N were collected from four separate districts in 2015. Four of the ten *pfdhps* 437G (i.e. triple or quadruple mutant) mutant samples were collected in 2015 and six in 2016. Six of the ten samples came from Ujjain but all from different parts of Ujjain. There were no significant temporal or spatial trends.

### *Pfcrt* K76T and *pfmdr1* N86Y

*Pfcrt* 76T was detected in 100% (102/102) of samples. *Pfmdr1* N86 was found in 72% (83/115) and *pfmdr1* 86Y in 28% (32/115) of samples. *Pfmdr1* N86 was found in 10/10 samples with the *pfdhps* 437G allele and in 59/89 (66%) samples with the *pfdhps* 437A allele (p < 0.001).

### K13 propeller region

The *P. falciparum k13* propeller region was successfully sequenced in 92 samples collected in 2009 and 2010 prior to the introduction of ASP and in 87 samples collected in 2015 and 2016. Sensitive haplotypes only were found in all (n = 179) successfully sequenced samples.

## Discussion

This is the second characterization of key anti-malarial drug resistance associated genetic polymorphisms in *P. falciparum* field isolates in Ujjain, Madhya Pradesh, Central India. Samples were collected 3 and 4 years after the implementation of ASP as first-line anti-malarial in Ujjain (2012) and 5 years after the first base-line study was conducted. From these data, the frequency of in vivo chloroquine and SP resistance in the study area and an indication of the speed at which resistant genotypes accumulate can be inferred.

The most encouraging finding was that the frequency of the previously identified key SP resistance associated alleles had not increased significantly despite the presumed increased SP drug pressure. In the previous study, 90% (70/78) and 96% (75/78) of samples had *pfdhfr* 59**R** and 108 **N** mutations, respectively, compared to 96% in this study. Similarly, 100% (76/76) and 9% (7/77) of samples had the *pfdhps* 436**F** and 437**G** mutations, respectively in the previous study compared to 100% and 10% in the current study. Furthermore, the triple mutant *pfdhfr* 59**R**108**N** plus *pfdhps* 437**G** haplotype was also stable at a frequency of 10% in the current study compared to 8% (6/76) prior to introduction of ASP. As in the previous study no alleles at *pfdhfr* codons 51 or 164 nor at *pfdhps* codons 540, 581 or 613 that are associated with high levels of SP resistance were found. The results suggest that mutations associated with SP resistance have not accumulated rapidly since the introduction of ASP 3 to 4 years previously.

However, the majority of samples 68/83 had a double mutation (*pfdhfr* 59**R** + 108 **N**), triple mutations were found in 12% of samples and unlike the previous study a quadruple mutation (*pfdhfr* 50**R**59**R**108 **N** and *pfdhps* 437**G**) was found. Moreover, the triple *pfdhfr* 50**R**59**R**108 **N**, that was not seen prior to the introduction of ASP was seen in 5% of samples (4/87) possibly indicating that this haplotype is beginning to accumulate. In a more western part of Madhya Pradesh state, the *pfdhfr* 108 **N**, 59**R** and 51**I** frequencies were 80%, 57% and 32%, respectively and the frequency of triple *pfdhfr* mutations were 0%, 2%, and 3% in 2012, 2013 and 2014. The frequency of *pfdhps* double mutations were 0, 3 and 8.5% the same respective years [29]. The numbers are small but point in the same direction as our data possibly indicating that more resistant haplotypes are evolving in Madhya Pradesh. However, the risk of importing highly SP resistant *P. falciparum* is perhaps greater than the risk of local evolution as the frequency of *pfdhfr* + *pfdhps* with 6 or 7 mutations correlating to highly SP resistant parasites were 38% (85/226) in a study conducted in 2014–2016 only slightly further west, in West Bengal [2].

In the same West Bengal study various *pfdhfr* and *pfdhps* genotypes were tested for in vitro susceptibility [2]. Approximately half the parasites with *pfdhfr* 59**R**108**N** had IC50 values suggestive of pyrimethamine resistance. Samples with *pfdhps* 437**G** or 436**A** had sulfadoxine IC50 values ranging up to resistance level. Half the samples with 436**A**437**G** had IC50 values suggesting resistance. The effect of the *pfdhps* 436**F** mutation only on IC50 values was not assessed but has previously been shown to modulate sulfadoxine susceptibility [30]. The 82% frequency of *pfdhfr* 59**R**108**N** plus *pfdhps* 436F and the 11% frequency *pfdhfr* 59R108N plus *pfdhps* 436**F**437**G** thus suggests that the parasites are at least SP tolerant verging on resistant at our site. In line with these data, triple mutation *pfdhfr* 59**R**108**N **+ *pfdhps* 437**G** have been associated with SP treatment failure in India [31].

Similar to findings in western Madhya Pradesh and Southwestern India but unlike findings in Northeastern India and West Bengal there were no mutations in the *kelch*-*13* propeller domain suggesting that parasites remained artemisinin susceptible at the study site [2, 29, 32, 33]. ASP efficacy was 99.6% in the Madhya Pradesh study and 84% in the West Bengal study [2, 29]. The lack of mutations linked to delayed parasite clearance when treated with artemisinin or high degree of SP resistance suggests that the ASP efficacy at our site should be closer to the 99% found in Madhya Pradesh. The results thus support the continued use of ASP at the study site assuming continued direct or indirect monitoring of ASP efficacy.

In this study and in a previous report from the same study site *pfdhps* 436F was fixed [18]. Interestingly it was not noted further east in Madhya Pradesh but even further east in Orissa, the *pfdhps* **F**_436_A_437_K_540_A_581_A_613_ haplotype was found in 52% of samples [34]. Furthermore, the **F**_436_A_437_K_540_A_581_A_613_ and **F**_436_**G**_437_K_540_A_581_A_613_ haplotypes were found in 44% and 21% of samples at the same time as 33% carried the S_436_A_437_K_540_A_581_A_613_ haplotypes in study from Kolkata [35]. The 436F allele is thus not uncommon in India and its frequency varies.

The evolution of SP resistance in Asia has been shown to be ordered [12]. It appears to start with two initial mutations in *pfdhfr* (108**N** and then 59**R**), followed by two in *pfdhps* 437**G** and then either 540**E** or 581**G**. A third mutation then accumulates in each of *pfdhfr* and *pfdhps* [12]. In Ujjain, *pfdhfr* 50**R** appears to be developing in *P. falciparum* that already have *pfdhfr* 108**N**59**R** and *pfdhps* 436**F** concurrently with development of the *pfdhfr* (108**N** and then 59**R**) *pfdhps* 436**F**437**G** haplotype at our site. The numbers are small and no conclusions should be made. However, the *pfdhps* 436F allele has been shown to modulate sulfadoxine susceptibility [30] and was not detected in the study assessing the ordered evolution of SP resistance causing SNPs [12]. Its potential effect in the ordered accumulation of SNPs may thus be significant thought this has not been shown.

The 100% frequency of *pfcrt* 76T is significantly higher compared to the 96% (80/84) frequency prior to introduction of ASP (fishers exact p = 0.04). Similarly, though not significantly the *pfmdr1* 86Y frequency increased from 16% (13/83) prior to ASP introduction to 28% in the current study. This clearly indicates that chloroquine is not a viable treatment option. The *pfcrt* 76T and *pfmdr1* 86Y alleles are linked to lower lumefantrine IC 50 values suggesting that artemether–lumefantrine is a good second-line treatment in the case of ASP failure.

Finally, the poor PCR outcome in DNA extracted from RDTs is in line with other studies indicating the difficulty of extracting sufficient DNA [36]. Optimally blood for this type of survey should, therefore, be collected by other means so as not to introduce potential bias such as not being able to analyse samples with low parasite density.

## Conclusions

No *k13* SNPs were found and the frequency of SNPs associated with SP treatment failure was virtually unchanged 3–4 years after compared to before the introduction of ASP in Ujjain, Madhya Pradesh, Central India. However, a single quadruple mutation was found and a novel *pfdhfr* triple mutation was found in 5% of samples. The results support the continued use of ASP at the study site but indicate that continuous monitoring is necessary.

## Data Availability

The datasets used and/or analysed during the current study are available from the corresponding author on reasonable request.

## Abbreviations

AL: Artemether–lumefantrine
ASP: Artesunate plus sulfadoxine–pyrimethamine
PCR: Polymerase chain reaction
*Pfcrt P. falciparum*: Chloroquine resistance transporter
*Pfdhfr P. falciparum*: Dihydrofolate reductase
*Pfdhps P. falciparum*: Dihyropteroate synthase
*Pfmdr1 P. falciparum*: Multi drug resistance gene 1
*K13*: *Kelch 13*
RFLP: Resitriction fragment length polymorphism
SP: Sulfadoxine–pyrimethamine
